# The Potential Biomarkers to Identify the Development of Steatosis in Hyperuricemia

**DOI:** 10.1371/journal.pone.0149043

**Published:** 2016-02-18

**Authors:** Yong Tan, Xinru Liu, Ke Zhou, Xiaojuan He, Cheng Lu, Bing He, Xuyan Niu, Cheng Xiao, Gang Xu, Zhaoxiang Bian, Xianpeng Zu, Ge Zhang, Weidong Zhang, Aiping Lu

**Affiliations:** 1 Institute of Basic Research in Clinical Medicine, China Academy of Chinese Medical Sciences, Beijing, China; 2 School of Pharmacy, Second Military Medical University, Shanghai, China; 3 Second Affiliated Hospital, Hunan University of Chinese Medicine, Changsha, China; 4 Institute for Advancing Translational Medicine in Bone & Joint Diseases, School of Chinese Medicine, Hong Kong Baptist University, Hong Kong, China; 5 China-Japan Friendship Hospital, Beijing, China; 6 E-Institutes of Shanghai Municipal Education Commission, Shanghai, China; National Research Council of Italy, ITALY

## Abstract

Hyperuricemia (HU) often progresses to combine with non-alcoholic fatty liver disease (NAFLD) in the clinical scenario, which further exacerbates metabolic disorders; early detection of biomarkers, if obtained during the HU progression, may be beneficial for preventing its combination with NAFLD. This study aimed to decipher the biomarkers and mechanisms of the development of steatosis in HU. Four groups of subjects undergoing health screening, including healthy subjects, subjects with HU, subjects with HU combined with NAFLD (HU+NAFLD) and subjects with HU initially and then with HU+NAFLD one year later (HU→HU+NAFLD), were recruited in this study. The metabolic profiles of all subjects’ serum were analyzed by liquid chromatography quadruple time-of-flight mass spectrometry. The metabolomic data from subjects with HU and HU+NAFLD were compared, and the biomarkers for the progression from HU to HU+NAFLD were predicted. The metabolomic data from HU→HU+NAFLD subjects were collected for further verification. The results showed that the progression was associated with disturbances of phospholipase metabolism, purine nucleotide degradation and Liver X receptor/retinoic X receptor activation as characterized by up-regulated phosphatidic acid, cholesterol ester (18:0) and down-regulated inosine. These metabolic alterations may be at least partially responsible for the development of steatosis in HU. This study provides a new paradigm for better understanding and further prevention of disease progression.

## Introduction

Hyperuricemia (HU) is a common disease characterized by the presence of elevated blood uric acid levels, which is usually due to specific genetic variation, renal urate underexcretion and renal urate overload [1, 2]. Growing evidences also demonstrated that HU is associated with unhealthy lifestyle and dietary habits that are mainly represented by a poor diet with an excessive intake of purine nucleotides, protein, alcohol, etc. [3]. HU is regarded as an important predictive risk factor for non-alcoholic fatty liver disease (NAFLD) which is characterized by the presence of fat droplets in hepatocytes without alcohol consumption, representing a spectrum of hepatic injuries, ranging from simple steatosis to non-alcoholic steatohepatitis [4–7]. Further studies explored that HU often progress to combine with NAFLD in the clinical scenario (HU+NAFLD), which not only further exacerbates metabolic disorders but may lead to histological liver damage [8–10]. Early detection of biomarkers, if obtained during the HU progression, may be beneficial for preventing its combination with NAFLD.

The pathogenesis of HU and NAFLD are both associated with the metabolic disorder of human body substances that involve many different molecules [11]. It is difficult to detect the complex groups of these molecules using conventional analytical techniques [12, 13]. Metabolomic analysis can provide detailed evidence for an in-depth study of the small biochemical present in a biological sample, bringing enormous opportunities for improved detection of biomarker discovery in a holistic context [14–16]. High-resolution mass analyzers (e.g., time-of-flight, TOF) can be used to obtain accurate mass measurements for determining the elemental compositions of metabolites [17]. Combining these analyzers with conventional MS/MS provides useful additional structural information for the identification of metabolites [18–20].

In the present study, a metabolomics-based liquid chromatography quadruple time-of-flight mass spectrometry (LC-Q-TOF-MS) technique with a pattern-recognition approach was employed to demonstrate the serum metabolic characteristics. We compared the metabolic biomarkers of HU and HU+NAFLD and predicted the biomarkers for the progression from HU to HU+NAFLD. Additionally, we collected metabolomic data from the subjects who suffered first from HU and then one year later from HU+NAFLD for further verification. We aimed to unveil the sensitive, reliable biomarkers responsible for the development of steatosis in HU.

## Materials and Methods

### Study Populations

**Fig 1:**
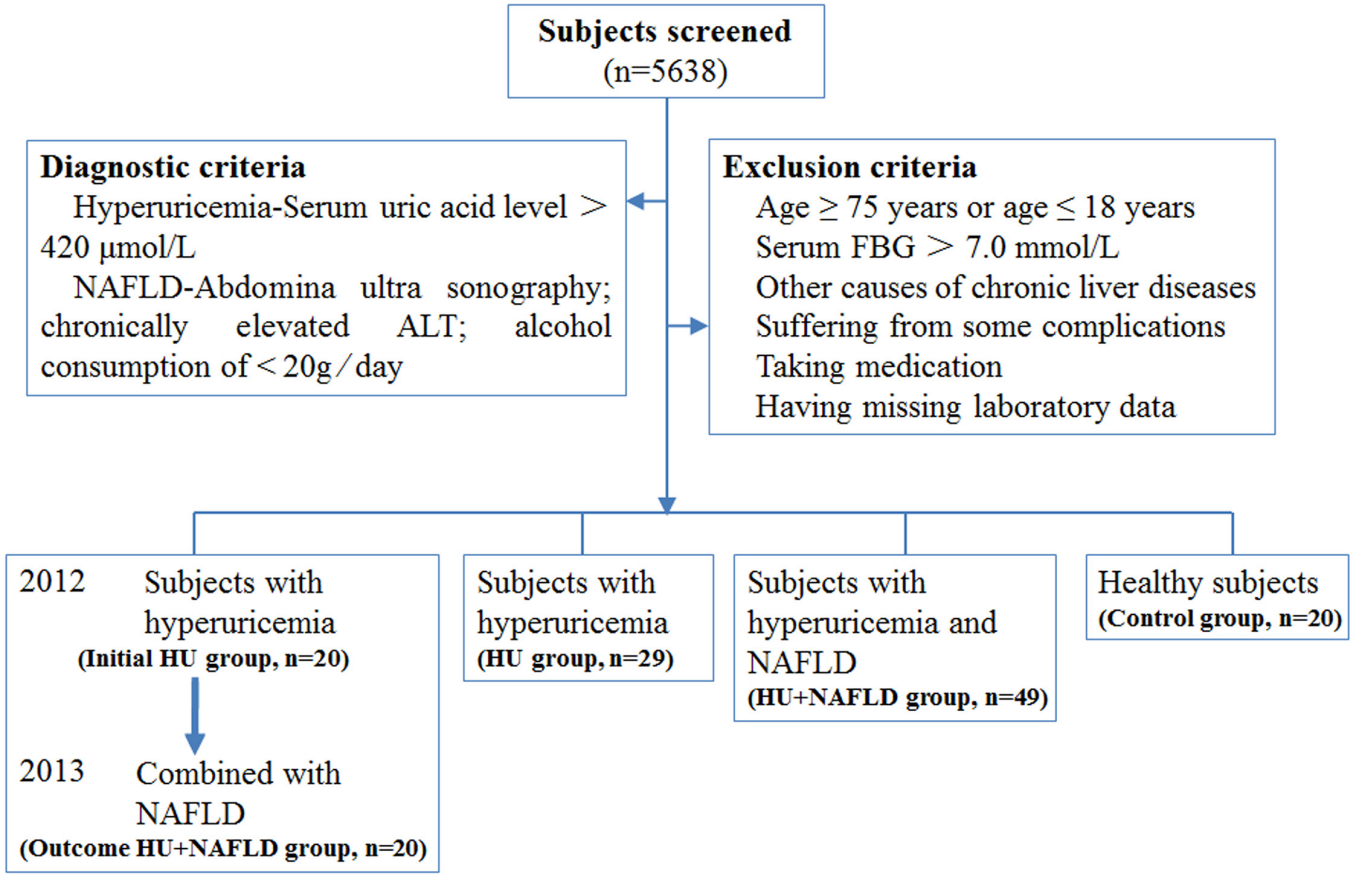
Subjects enrolled in the study.

In total, 5638 individuals (4082 males and 1556 females) underwent health screening at Hangxin Hospital, Beijing, China, in 2012 and 2013. Definitions of hyperuricemia were high fasting serum uric acid (SUA) (>420μmol/L) at physiological temperature (37°C) and neutral pH. The diagnosis of NAFLD was based on the findings of abdominal ultrasound, clinical chemistry according to conventional criteria and alcohol consumption of <20g⁄day in the last year. Some subjects were excluded for the following reasons: age≥75 years or age≤18 years; fasting blood glucose (FBG)>7.0 mmol/L or a history of diabetes mellitus; other causes of chronic liver diseases or mixed aetiologies (hepatitis C, hepatitis B, autoimmune liver disease, Wilson’s disease, hemochromatosis and α1-antitrypsin deficiency); complications such as cardiovascular and cerebrovascular diseases, nephropathy, dyslipidemia, morbid obesity (body mass index: BMI≥40 kg/m^2^); intake of antihypouricemic (allopurinol, probenecid, sulfinpyrazone and benzbromarone) and lipotropic medications (polyene phosphatidyl choline, ursodeoxycholic acid, inosine and reduced glutathione). Consequently, 118 subjects comprised the cohort for this study, including 20 healthy subjects (Control group: 13 males and 7 females), 29 subjects with hyperuricemia (HU group: 19 males and 10 females), 49 subjects with HU+NAFLD (HU+NAFLD group: 33 males and 16 females), as well as 20 subjects with hyperuricemia in 2012 (Initial HU group: 13 males and 7 females) and HU+NAFLD one year later (Outcome HU+NAFLD group) (**Fig 1**). This study was approved by the Ethics Committee at the Institute of Basic Research in Clinical Medicine, China Academy of Chinese Medical Sciences and was conducted according to the standards of the Declaration of Helsinki. Written informed consent was obtained from the participants.

### Questionnaire

All participants were asked to fill out a questionnaire regarding their medical history, drug usage, alcohol intake and health-related behavior under the guidance of physicians. The questions on alcohol intake included the frequency of alcohol consumption per week and the usual amount that was consumed.

### Blood sampling and biochemical test

Fasting blood samples were drawn via venipuncture from the study participants by clinical nurses. After storage for 2 h at 4°C, the blood samples were centrifuged at 3500×g for 15 min. The obtained serum was divided into two parts: one part was used for the measurement of uric acid (UA), fasting blood glucose (FBG), total cholesterol (TC), triglyceride (TG), high- and low-density lipoprotein cholesterol (HDL-C, LDL-C), blood urea nitrogen (BUN), creatinine (CRE), aspartate aminotransferase (AST) and alanine aminotransferase (ALT) concentrations according to the manufacturer’s instructions for the respective commercial test kits. The remaining 100 μL serum was added to 200 μL of methanol, and the mixture was vortexed for 30 s. After centrifugation at 9560×g for 10 min at 4°C, the supernatant was stored at -80°C for LC/MS analysis.

### Ultrasonography

Hepatic ultrasonography was performed by a well-trained ultrasonographer. The characteristic ultrasonographic features that were used to diagnose hepatic steatosis included evidence of diffuse hyperechogenicity of the liver relative to the kidneys, ultrasound beam attenuation and poor visualization of intrahepatic vessel borders and diaphragm [21, 22].

### LC-Q-TOF-MS analysis

LC-Q-TOF-MS analysis was performed by using an Agilent-1200 LC system coupled with an electrospray ionization (ESI) source (Agilent Technologies, Palo Alto, CA, USA) and an Agilent-6520 Q-TOF mass spectrometer. Separation of all samples was performed on an Eclipse plus C18 column (1.8 μm, 3.6 mm×100 mm, Agilent) with a column temperature set at 45°C. The flow rate was 0.3 mL/min, and the mobile phase consisted of ultrapure water with 0.1% formic acid and acetonitrile. The following gradient program was used: 2% acetonitrile for 0–1.5 min; 2–100% acetonitrile for 1.5–13 min; a wash with 100% acetonitrile for 13–16 min; a re-equilibration step for 5 min. The sample injection volume was 2 μL.

Mass detection was operated in the positive ion mode with the following setting: drying gas (N2) flow rate, 8 L/min; gas temperature, 330°C; pressure of nebulizer gas, 35 psig; Vcap, 4000 V; fragmentor, 160 V; skimmer, 65 V; scan range, m/z 50–1200. All analyses were acquired using the instrument mass spray to ensure accuracy and reproducibility. Leucine enkephalin was used as the instrument reference mass (m/z 556. 2771) at a concentration of 50 fmol/μL with a flow rate 40 μL/min. The MS/MS analysis was acquired in targeted MS/MS mode with collision energy from 10 V to 40 V.

### Sequence analysis

The pooled QC sample was analyzed at the beginning, at the end and randomly throughout the analytical run to monitor the stability of the sequence analysis. The typical batch sequence of serum samples consisted of the consecutive analysis of 1 QC serum sample (at the beginning of the study), followed by 6 unknown serum samples, and then 1 QC serum sample, before running another 6 unknown serum samples, etc. Meanwhile, the samples were analyzed in a random order as per normal good practice. An identical sequence was repeated to complete the total set of injections (n = 104, including QCs) analyzed in less than 1 day per mode, as described in previous studies.

### Data processing and statistical analysis

The LC–MS raw data were exported by the Agilent Mass Hunter Qualitative Analysis Software (Agilent Technologies, Palo Alto, CA, USA). The data of each sample were normalized to the total area to correct for the MS response shift between injections due to any possible intra- and inter-day variations. The sum of the ion peak areas within each sample was normalized to 10,000. Partial least squares discriminant analysis (PLS-DA) was used for the metabolic profile analysis. The differentiation performance was validated by the area under the curve (AUC) of receiver operating characteristic (ROC) curves. Multivariate analysis was performed by the SIMCA-P version 11 software (Umetrics AB, Umeå, Sweden). SAS 9.1.3 statistical package (order no. 195557) was used for the statistical analysis. Chi-square test was used for analysis of attribute data. The measurement data obtained showed a normal distribution. Variance analysis was used for comparisons between multiple groups. P<0.05 was considered statistically significant.

### IPA analysis

The analyses of the networks, bio-functions and canonical pathways were conducted by using the Ingenuity Pathway Analysis system (IPA, Ingenuity oR Systems, http://www.ingenuity.com) for the candidate metabolites, to gain insight into the typical metabolic alterations associated with the biomarkers and the mechanism relevant to the progression from HU to HU+NAFLD.

### Prediction of metabolites indication ability

Human enzyme-metabolite interaction (EMI) data were downloaded from the HMDB database. Human protein-protein interaction (PPI) data were collected from the HPRD database and BioGRID database. EMIs and PPIs supported by at least one wet experiment study were considered confident and were selected for further analysis. Ultimately, 452985 EMIs and 304705 PPIs were used in this analysis. It has been hypothesized that changes in metabolites represent changes in the enzymes that participate in catalyzing the metabolites. Because the changes in enzymes are the results of deregulating up-stream pathways in diseases, the metabolites may be used to indicate the internal molecular abnormal state of the disease. Representative value (RV) is defined as the power of the metabolite to reflect the abnormality of the disease. RV uses the fold change of the metabolite, number of enzymes catalyzing the metabolite and the importance of every enzyme to evaluate the indicative ability of the metabolite for the disease. RV is calculated as follows:

$${\text{RV}}_{m}=\frac{FC_{m}\sum_{i=1}^{n_{e}}EP_{i}}{\sum_{j=1}^{n_{m}}(FC_{m_{j}}\sum_{i=1}^{n_{e}}EP_{i})}$$

RV_m_ is the representative value of the metabolite m;

EP_i_ is the network power of the enzyme i that participates catalyzing the metabolite m. The network power is estimated by the protein-protein interaction (PPI) network degree;

n_e_ is the number of enzymes participating in catalyzing the metabolite m;

FC_m_ is the fold change value of the metabolite m in the disease compared with the normal state;

n_m_ is the number of deregulated metabolites in the disease.

Because the abnormality of the disease may be measured by metabolites, the metabolites can be used to indicate the progression from HU to HU+NAFLD. The progression indication value is defined as the power of the metabolite to indicate the progression. It is calculated as follows:

$${\text{TI}}_{m}=\frac{{\text{RV}}_{m}^{HU+NAFLD}-{\text{RV}}_{m}^{HU}}{\sum_{i=1}^{n_{m}}({\text{RV}}_{m_{i}}^{HU+NAFLD}-{\text{RV}}_{m_{i}}^{HU})}$$

TI_*m*_ is the progression indication value of the metabolite m, which reveals the ability of m to indicate the progression from HU to HU+NAFLD;

${\text{RV}}_{m}^{HU+NAFD}$ is the representative value of the metabolite m in HU+NAFLD; ${\text{RV}}_{m}^{HU}$ is the representative value of the metabolite m in HU;

*n*_*m*_ is the number of deregulated metabolites in HU+NAFLD and HU.

## Results

### Baseline characteristics of the study subjects

The clinical and biochemical characteristics of the enrolled subjects are shown in **Table 1**. Serum uric acid was significantly higher in the HU, HU+NAFLD, initial HU and outcome HU+NAFLD groups than in the healthy controls. In addition, serum ALT was significantly higher in the HU+NAFLD and outcome HU+NAFLD groups than in the healthy controls. The other data exhibited no statistically significant differences.

**Table 1:** Clinical and biochemical characteristics of each group of subjects.

Clinical indicator	HU	HU+NAFLD	Initial HU	Outcome HU+NAFLD	Healthy control
**Gender (M / F)**	19 / 10	33 / 16	13 / 7	13 / 7	13 / 7
**Age (year)**	49.28±8.61	48.98±14.99	48.55±14.04	49.55±14.04	49.42±11.50
**BMI (kg/m**^**2**^**)**	23.16±4.15	23.52±4.12	24.01±3.30	23.88±2.93	23.17±3.05
**SBP (mmHg)**	129.45±7.13	128.43±7.63	128.15±4.99	128.55±7.33	124.87±11.88
**DBP (mmHg)**	77.41±5.97	77.92±4.36	77.05±3.15	76.00±5.30	76.08±5.69
**FBG (mmol/L)**	5.37±0.29	5.34±0.26	5.16±0.46	5.38±0.34	5.28±0.38
**TG (mmol/L)**	1.31±0.49	1.17±0.25	1.13±0.25	1.10±0.20	1.13±0.44
**TC (mmol/L)**	4.22±0.88	4.15±0.93	4.21±0.89	4.07±0.93	4.16±0.86
**CHO (mmol/L)**	4.26±0.46	4.40±0.89	4.32±0.55	4.70±0.71	4.48±0.70
**HDL (mmol/L)**	1.18±0.34	1.26±0.30	1.25±0.28	1.18±0.29	1.22±0.30
**LDL (mmol/L)**	2.71±0.52	2.68±0.50	2.58±0.53	2.59±0.55	2.66±0.52
**BUN (mmol/L)**	5.08±0.97	5.18±0.95	5.29±1.00	5.34±1.01	5.2±0.98
**CRE (mmol/L)**	70.74±14.77	70.49±14.42	69.62±13.54	70.62±14.51	70.32±14.09
**UA (μmol/L)**	424.28±46.23**	436.69±32.55**	437.6±29.16**	448.75±39.82**	294.69±45.38
**AST (U/L)**	22.49±5.64	24.32±7.11	24.53±5.86	24.90±6.67	23.46±6.54
**ALT (U/L)**	20.69±6.32	24.96±8.95**	20.15±6.51	25.61±8.36**	19.61±6.83

Note: The comparisons of clinical indicators among HU, HU+NAFLD, initial HU, outcome HU+NAFLD and Control subjects. Continuous variables were analyzed by unpaired t-test, and the data were expressed as the mean ± SD when appropriate (95% CI). HU, HU+NAFLD, initial HU and outcome HU+NAFLD vs. Control, respectively:

*p < 0.05,

**p < 0.01. Count variables were analyzed by Chi-square test.

### Assessment of the repeatability and stability of the LC-Q-TOF-MS method

Extracts from six aliquots of a random blood sample were continuously injected to evaluate repeatability [17, 23]. Five common extracted ion chromatograms (EICs) shared by these injections were selected according to their different chemical polarities and m/z values. The relative standard derivations (RSDs) of these peaks were 4.13–13.13% for peak areas and 0.04–0.98% for retention times.

The LC-MS system stability for the large-scale sample analysis was demonstrated by the test of pooled QC samples. The principal components analysis (PCA) result showed that the QC samples were tightly clustered. Moreover, the peak areas, retention times and mass accuracies of five selected EICs in five QC samples also showed good system stability. RSDs of the five peaks were 4.94–14.88% for peak areas, 0.03–1.10% for retention times and 0.14E-04%–0.76E-04% for mass accuracies. The results indicated that the large-scale sample analysis had no apparent effect on the reliability of the data.

### Identification of the differential metabolites and analysis of the biological association networks in HU and HU+NAFLD

**Fig 2:**
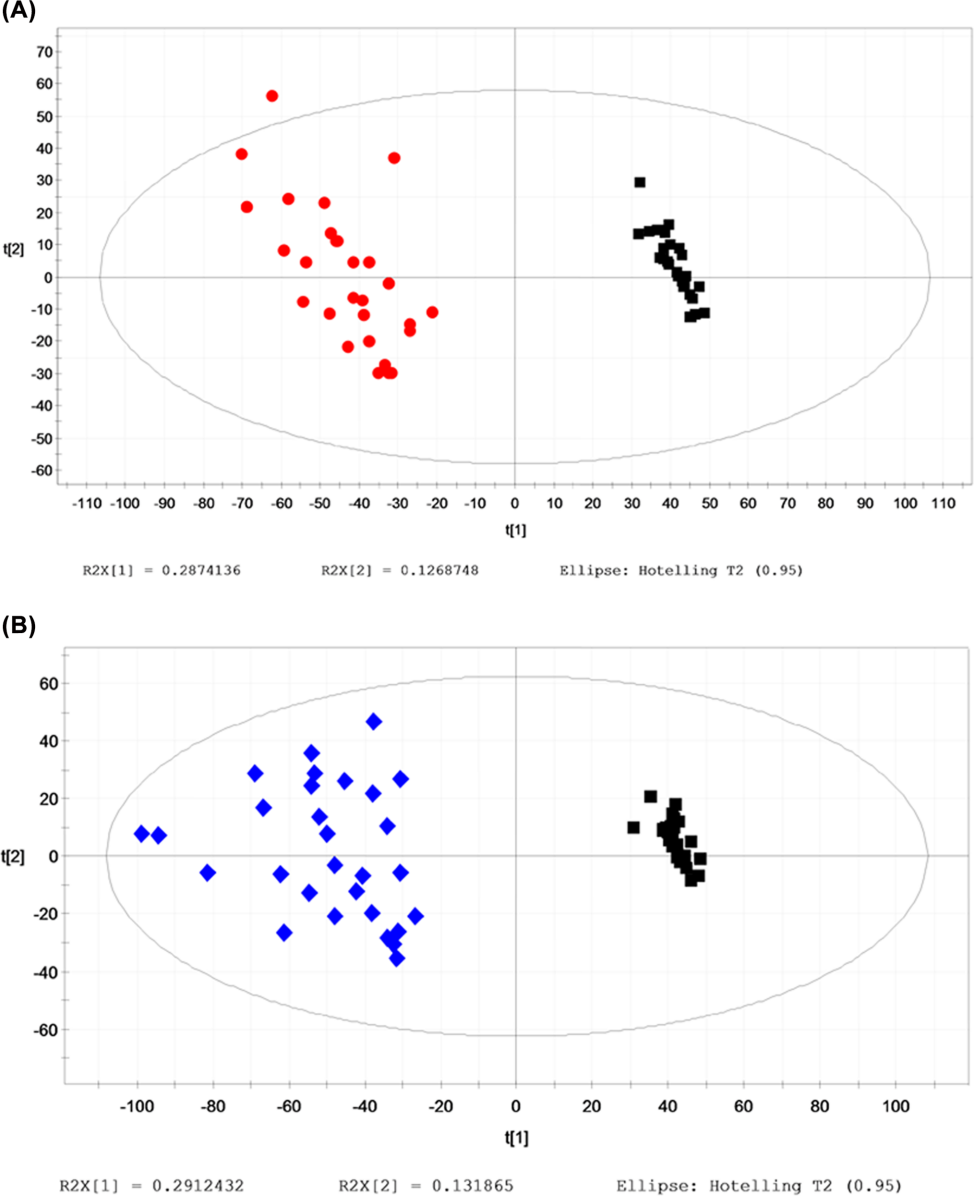
Multiple pattern recognition of the serum metabolites. (A) In Control and HU: PLS-DA score plot (n = 49), Control (■), Hyperuricemia (●); (B) In Control and HU+NAFLD: PLS-DA score plot (n = 69), Control (■), HU+NAFLD (◆).

Typical base peak chromatograms (BPCs) of the serum samples were obtained from the Control, HU and HU+NAFLD subjects. Based on the metabolic changes in those subjects as revealed by BPCs, we adopted the multiple pattern recognition methods of PLS-DA. This approach facilitated the classification of the metabolic phenotypes and enabled us to further identify the differential metabolites. The score plots demonstrated an obvious separation between the Control, HU and HU+NAFLD groups, as illustrated in **Fig 2A and 2B**.

**Fig 3:**
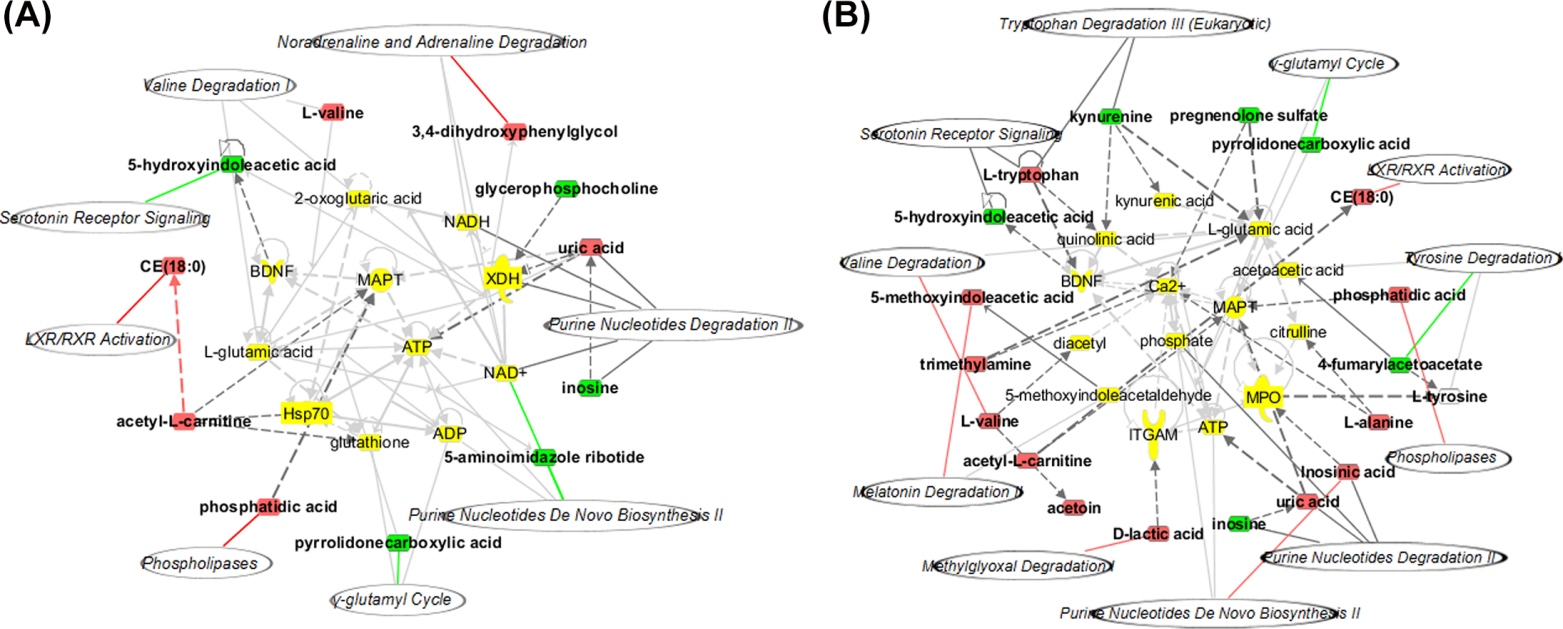
Biological association network related to the identified differential metabolites. Molecules in the network are represented as nodes, and the biological relationship between two nodes is represented as a line. Note that the colored symbols represent the metabolites and pathways that occurred in the findings, while the transparent entries are molecules from the Ingenuity Knowledge Database. Red symbols represent up-regulated metabolites, green symbols represent down-regulated metabolites, and italicized symbols represent canonical pathways that are related to the identified specific metabolites. Solid lines between molecules indicate a direct physical relationship between the molecules, while dotted lines indicate indirect functional relationships. (A) HU network, (B) HU+NAFLD network.

Compared with those in the Control subjects, 11 metabolites were identified in the HU subjects (**Table 2**), and 18 metabolites were identified in the HU+NAFLD subjects (**Table 3**). To further understand the correlation between the identified metabolites, the analyses were performed using IPA software, providing the identification of biological association networks and canonical pathways (**Fig 3A and 3B**). **Fig 3A** shows the merged network of the metabolites identified in HU. These metabolites were correlated with the five top canonical pathways, including purine nucleotide degradation II, liver X receptor/retinoic X receptor (LXR/RXR) activation, phospholipases, serotonin receptor signaling and purine nucleotides de novo biosynthesis II (**S1 Table**). Associated network functions include lipid metabolism, nitric oxide synthesis, carboxylic acid transport, and purine nucleotide metabolism. **Fig 3B** shows the merged network of the metabolites identified in HU+NAFLD. The five top canonical pathways that are correlated with the identified metabolites include purine nucleotide degradation II, serotonin receptor signaling, tryptophan degradation III, phospholipases and LXR/RXR activation (**S2 Table**). Associated network functions include purine nucleotide metabolism, amino acid metabolism, lipid metabolism and energy production. The analyses revealed that four canonical pathways (purine nucleotide degradation II, phospholipases, serotonin receptor signaling and LXR/RXR activation) and two network functions (purine nucleotide metabolism and lipid metabolism) were closely related to the identified differential metabolites in both HU and HU+NAFLD.

**Table 2:** Identified differential metabolites in the HU serum.

n	tR (min)	Extract mass	Formula	Compound	Folder
**1**	13.49	687.4839	C_35_H_69_O_8_P	Phosphatidic acid	7.0949
**2**	2.02	268.0808	C_10_H_12_N_4_O_5_	Inosine	-7.8434
**3**	2.25	170.0579	C_8_H_10_O_4_	3,4-dihydroxyphenylglycol	4.9539
**4**	8.73	191.0582	C_10_H_9_NO_3_	5-hydroxyindoleacetic acid	-1.8216
**5**	1.76	295.0569	C_8_H_14_N_3_O_7_P	5-aminoimidazole ribotide	-1.5211
**6**	3.05	129.0426	C_5_H_7_NO_3_	Pyrrolidonecarboxylic acid	-1.6632
**7**	1.80	257.1028	C8H21NO6P	Glycerophosphocholine	-2.3768
**8**	2.16	117.0790	C5H11NO2	L-valine	1.3321
**9**	13.14	652.6158	C45H80O2	Cholesterol ester (CE) (18:0)	1.7558
**10**	2.02	168.0283	C_5_H_4_N_4_O_3_	Uric acid	1.6282
**11**	2.37	203.1157	C_9_H_18_NO_4_	Acetyl-L-carnitine	3.0658

Note: “Folder” refers to the “HU vs. Control” change value.

**Table 3:** Identified differential metabolites in the HU+NAFLD serum.

n	tR (min)	Extract mass	Formula	Compound	Folder
**1**	13.48	687.4839	C_35_H_69_O_8_P	Phosphatidic acid	11.2647
**2**	2.16	348.0471	C_10_H_13_N_4_O_8_P	Inosinic acid	4.3447
**3**	2.02	268.0808	C_10_H_12_N_4_O_5_	Inosine	-13.1045
**4**	2.59	208.0848	C_10_H_12_N_2_O_3_	Kynurenine	-3.7021
**5**	8.73	191.0582	C_10_H_9_NO_3_	5-hydroxyindoleacetic acid	-1.7946
**6**	3.23	204.0899	C_11_H_12_N_2_O_2_	L-tryptophan	1.8372
**7**	2.16	117.079	C_5_H_11_NO_2_	L-valine	1.3443
**8**	3.05	129.0426	C_5_H_7_NO_3_	Pyrrolidonecarboxylic acid	-1.6291
**9**	2.24	90.0317	C_3_H_6_O_3_	D-lactic acid	1.8559
**10**	13.14	652.6158	C_45_H_80_O_2_	CE (18:0)	2.8026
**11**	2.01	168.0283	C_5_H_4_N_4_O_3_	Uric acid	1.6742
**12**	3.31	200.0321	C_8_H_8_O_6_	4-fumarylacetoacetate	-3.0421
**13**	3.42	59.0735	C_3_H_9_N	Trimethylamine	5.4471
**14**	2.33	203.1157	C_9_H_18_NO_4_	Acetyl-L-carnitine	2.9672
**15**	1.72	89.0477	C_3_H_7_NO_2_	L-Alanine	2.2691
**16**	8.27	205.0739	C_11_H_11_NO_3_	5-methoxyindoleacetic acid	2.1157
**17**	2.78	88.0524	C_4_H_8_O_2_	Acetoin	3.4288
**18**	7.77	396.1970	C_21_H_32_O_5_S	Pregnenolone sulfate	-1.5579

Note: “Folder” refers to the “HU+NAFLD vs. Control” change value.

### Prediction of the metabolites and pathways responsible for the progression from HU to HU+NAFLD

To predict which metabolites were involved in the progression from HU to HU+NAFLD, we designed two novel indicators: RV represents the power of the metabolites to reflect the abnormality of the disease, and TI reveals the ability of the metabolites to indicate the progression. Using highly confident EMI and PPI data, as well as fold change data of the metabolites, we calculated RV and TI for every metabolite (see the details in the methods section). As shown in **Table 4**, 19 metabolites might involve in the progression from HU to HU+NAFLD, except for acetoin and pregnenolone sulfate in HU+NAFLD. From phosphatidic acid to L-valine, their corresponding TI values are reduced in sequence. Phosphatidic acid is the most representative metabolite in both HU and HU+NAFLD. Moreover, phosphatidic acid is the best metabolite to indicate the progression (**Table 4**).

**Table 4:** Progression indication ability of the identified differential metabolites.

n	Metabolite	RV		TI
		HU	HU+NAFLD	
**1**	Phosphatidic acid	0.6564	0.3867	0.3702
**2**	CE (18:0)	0.1945	0.2292	0.2874
**3**	Inosine	0.1353	0.2115	0.1383
**4**	3,4-dihydroxyphenylglycol	0.0533	0	0.0648
**5**	Kynurenine	0	0.0411	0.0488
**6**	Glycerophosphocholine	0.0224	0	0.0198
**7**	L-tryptophan	0	0.0197	0.0146
**8**	5-Aminoimidazole ribotide	0.0242	0	0.0126
**9**	D-lactic acid	0	0.0106	0.0079
**10**	5-hydroxyindoleacetic acid	0.0338	0.0276	0.0064
**11**	Trimethylamine	0	0.0047	0.0062
**12**	Inosinic acid	0	0.0103	0.0062
**13**	4-fumarylacetoacetate	0	0.0052	0.0057
**14**	Pyrrolidonecarboxylic acid	0.0231	0.0186	0.0049
**15**	Acetyl-L-carnitine	0.0063	0.0041	0.0029
**16**	L-alanine	0	0.0015	0.0014
**17**	Uric acid	0.0091	0.0087	0.0003
**18**	5-methoxyindoleacetic acid	0	0.0003	0.0003
**19**	L-valine	0.0205	0.0193	0.0002

RV is the power of the metabolite to reflect the abnormal state in the disease. TI is the progression indication value of the metabolite, which reveals the ability of the metabolite to indicate the progression from HU to HU+NAFLD.

**Fig 4:**
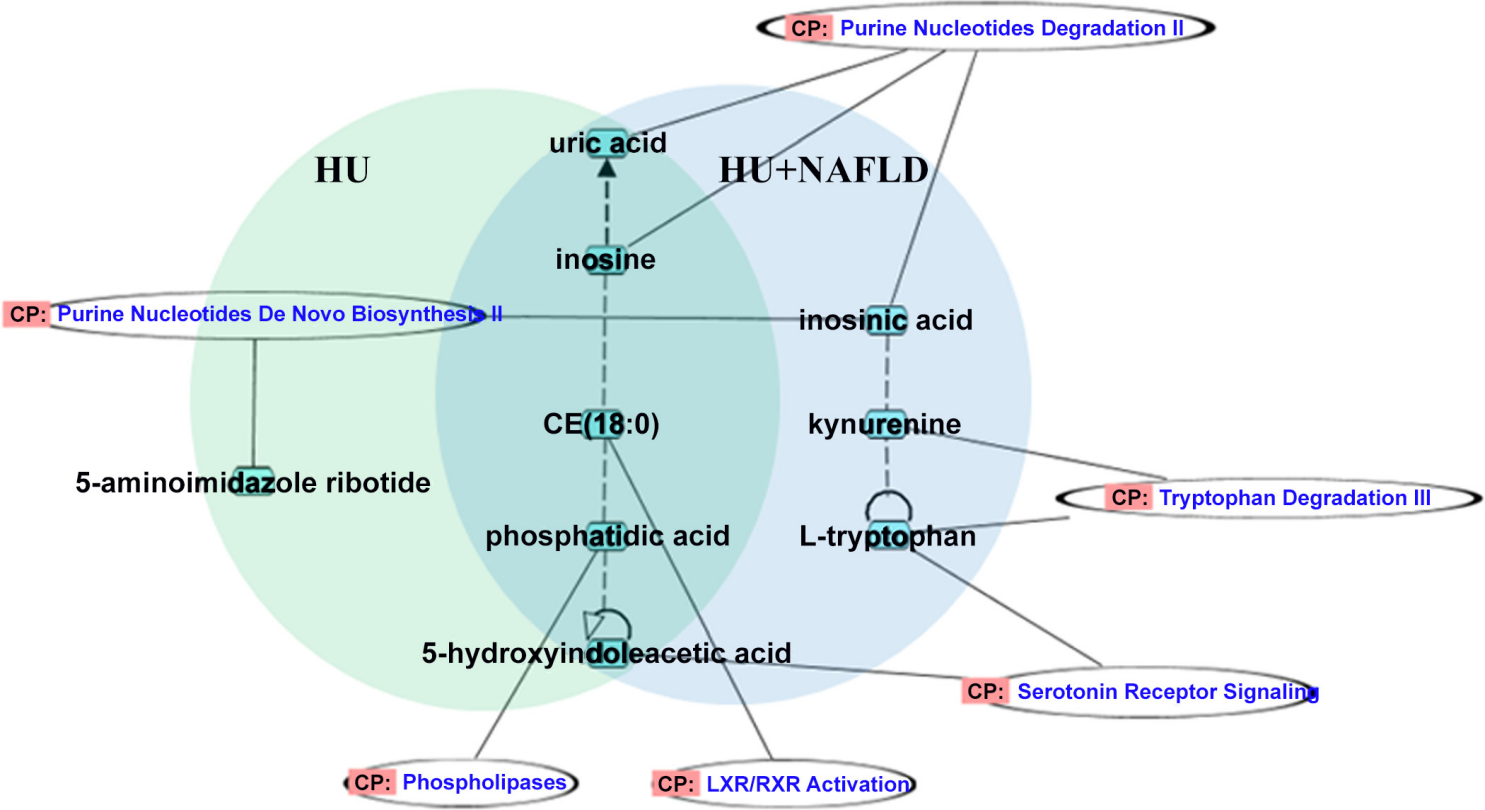
The identified differential metabolites and pathways responsible for the progression from HU to HU+NAFLD.

The correlation between these metabolites was further explored through IPA. The results showed that these metabolites could constitute a network and were associated with certain biological pathways, mainly including purine nucleotide degradation and biosynthesis related to inosine, uric acid, inosinic acid and 5-aminoimidazole ribotide. The metabolites were also related to tryptophan degradation III with kynurenine and L-tryptophan, serotonin receptor signaling with 5-hydroxyindoleacetic acid and L-tryptophan, phospholipases related to phosphatidic acid, and LXR/RXR activation in relation to cholesterol ester (CE) (18:0) (**Fig 4**and **S3 Table**).

**Fig 5:**
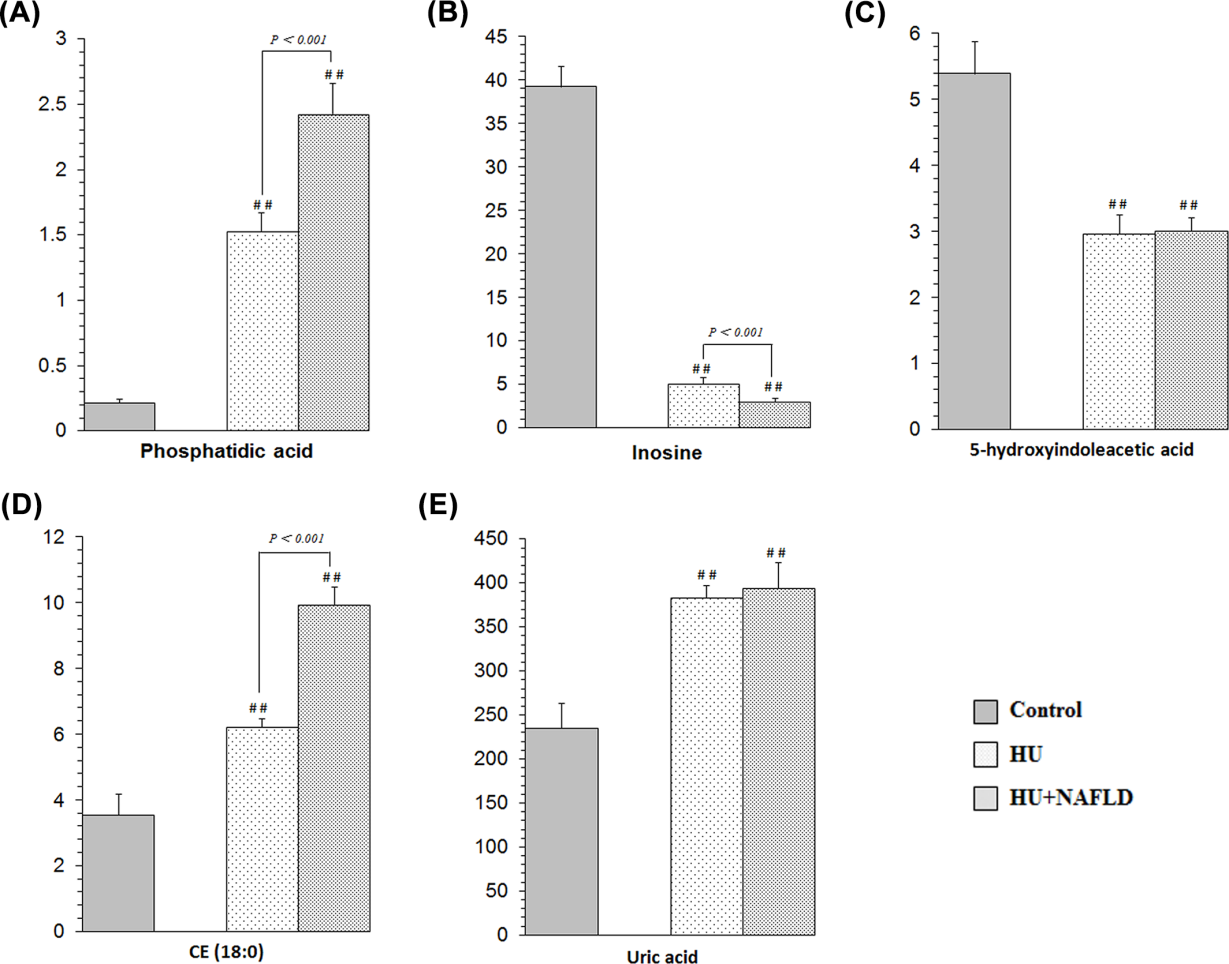
Comparison of the peak area intensities of the common metabolites in the progression from HU to HU+NAFLD. Significant difference from the Control: # p < 0.05, ## p < 0.01. HU+NAFLD vs. HU: when p < 0.05, the specific p valve is marked in the histograms. (A) Phosphatidic acid, (B) Inosine, (C) 5-hydroxyindoleacetic acid, (D) CE (18:0), (E) Uric acid.

As shown in **Fig 4**, there were five common metabolites in HU and HU+NAFLD. To explore their serum level change in the progression from HU to HU+NAFLD, an analysis of variance was used for the comparison between HU and HU+NAFLD. The results demonstrated that the phosphatidic acid and CE (18:0) levels in HU were lower than those in HU+NAFLD; by contrast, the inosine levels in HU were higher than those in HU+NAFLD (**Fig 5**).

### Verification of the metabolites and pathways related to the progression from HU to HU+NAFLD

**Fig 6:**
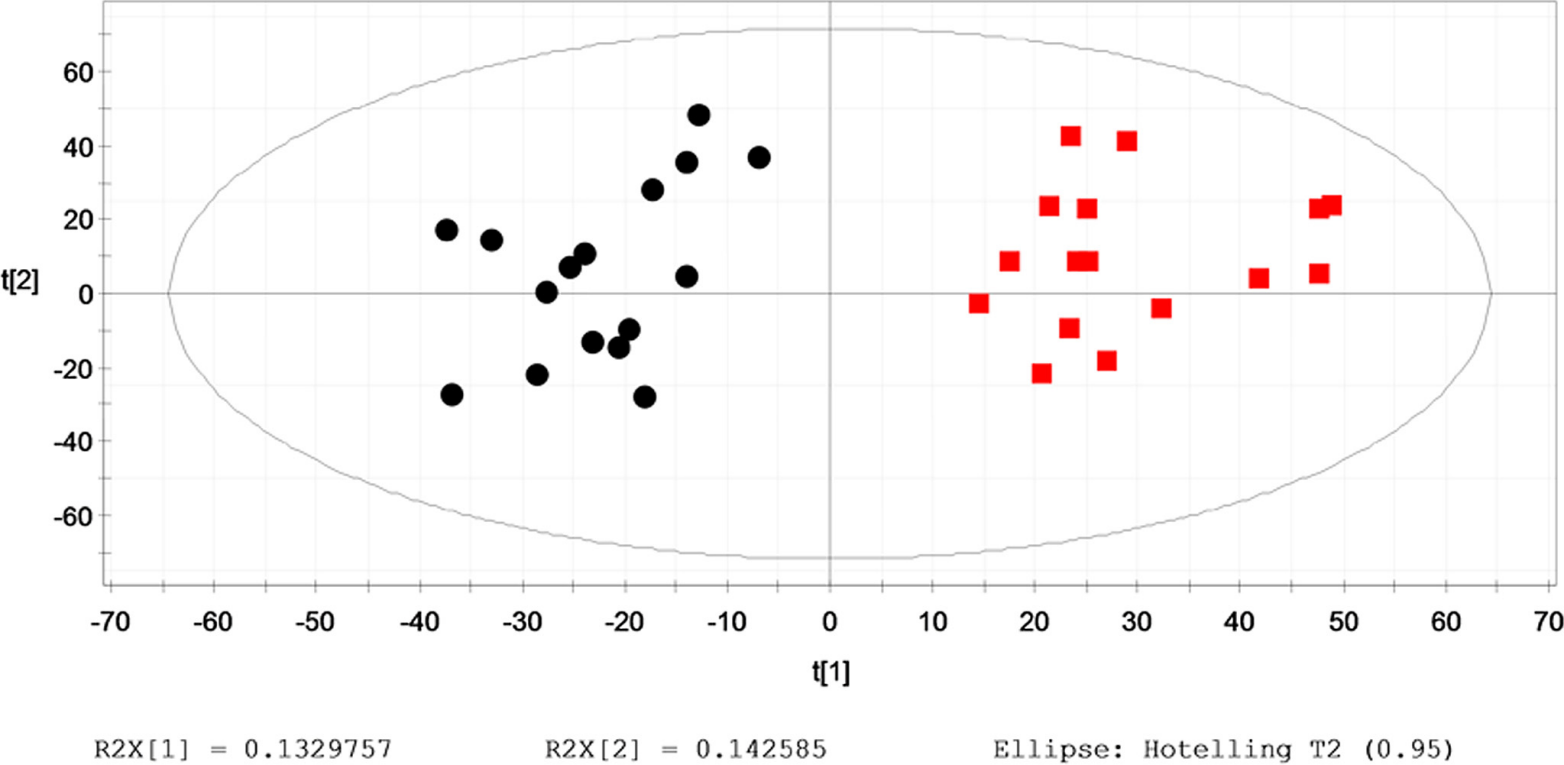
Multiple pattern recognition of serum metabolites in outcome HU+NAFLD vs. initial HU. PLS-DA score plot (n = 40). initial HU (●); outcome HU+NAFLD (■).

To verify the predicted metabolites and pathways responsible for the progression from HU to HU+NAFLD, we obtained a follow-up data set from the subjects who had HU in 2012 and subsequently were identified with HU+NAFLD in 2013. Based on the metabolic changes within two years, we adopted the multiple pattern recognition methods. PLS-DA score plots showed obvious separation between initial HU and outcome HU+NAFLD, as illustrated in **Fig 6**. Furthermore, the differential metabolites possessed higher values of AUC (AUC = 0.94), suggesting an excellent clinical ability for the prediction.

**Fig 7:**
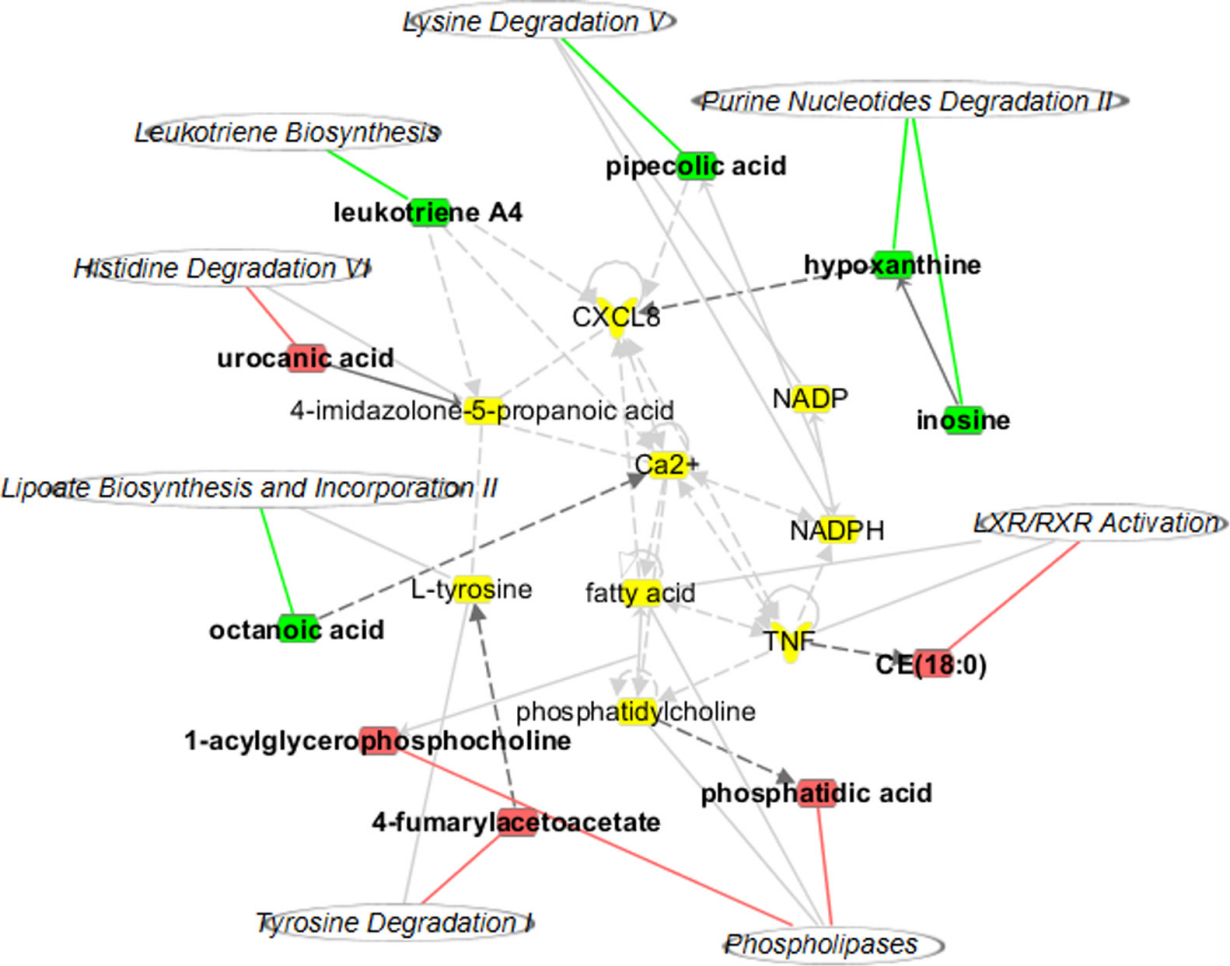
Biological association network related to the identified differential metabolites in HU→HU+NAFLD subjects. Molecules in the network are represented as nodes, and the biological relationship between two nodes is represented as a line. Note that the colored symbols represent metabolites and pathways that occur in the findings, while the transparent entries are molecules from the Ingenuity Knowledge Database. Red symbols represent up-regulated metabolites; green symbols represent down-regulated metabolites; italicized symbols represent canonical pathways that are related to the identified specific metabolites. Solid lines between molecules indicate a direct physical relationship between the molecules, while dotted lines indicate indirect functional relationships.

Compared with those in the initial HU subjects, 10 metabolites were identified in outcome HU+NAFLD (**Table 5**). The correlation between the metabolites was identified through IPA software. As shown in **Fig 7**, these metabolites are correlated with the five top canonical pathways, including phospholipases, purine nucleotide degradation II, LXR/RXR activation, lipoate biosynthesis and incorporation II and histidine degradation VI (**S4 Table**). Through the comparison between these metabolites and pathways and the predicted metabolites and pathways, we found that phosphatidic acid, inosine and CE (18:0) were their common metabolites. Accordingly, phospholipases, purine nucleotide degradation and LXR/RXR activation were their common biological pathways. Therefore, these metabolites and pathways were considered the biomarkers involved in the progression.

**Table 5:** Identified differential metabolites in outcome HU+NAFLD vs. initial HU.

n	tR (min)	Extract mass	Formula	Compound	Folder	RV
**1**	13.48	687.4839	C_35_H_69_O_8_P	Phosphatidic acid	2.5395	0.5473
**2**	14.80	144.115	C_28_H_57_O_9_P	1-acylglycerophosphocholine	2.4983	0.1875
**3**	2.02	268.0808	C_10_H_12_N_4_O_5_	Inosine	-1.9616	0.0851
**4**	2.32	136.0385	C_5_H_4_N_4_O	Hypoxanthine	-2.5162	0.0681
**5**	13.14	652.6158	C_45_H_80_O_2_	CE (18:0)	2.1786	0.0441
**6**	12.13	318.2195	C_20_H_30_O_3_	Leukotriene A4	-2.0423	0.0276
**7**	9.42	144.1150	C_12_H_20_O_2_	Octanoic acid	-2.1665	0.0201
**8**	3.33	200.0321	C_8_H_12_O_4_	4-fumarylacetoacetate	2.3581	0.0109
**9**	2.40	129.0790	C_22_H_41_NO	Pipecolic acid	-3.5472	0.0049
**10**	2.78	138.0429	C_6_H_6_N_2_O_2_	Urocanic acid	1.9961	0.0042

Note: Folder refers to the “outcome HU+NAFLD vs. initial HU” change value. RV is the power of the metabolite to reflect the abnormal state in the disease.

## Discussion

HU combined with NAFLD is the result of hyperuricemia progression. It not only exacerbates metabolic and hemodynamic diseases but also may lead to liver damage [8–10]. Deciphering the biomarkers and mechanism of the development of steatosis in HU is crucial to preventing disease progression. We are the first group to discover the serum biomarkers of the steatosis development based on serum metabolic profiles. Up-regulated phosphatidic acid and CE (18:0) and down-regulated inosine in the serum have been identified as the potential biomarkers and the corresponding altered phospholipases, purine nucleotide degradation and LXR/RXR activation may be partly responsible for the development of steatosis in HU.

Phosphatidic acid, as an important signal transducer, is involved in phospholipase metabolism [24–26]. The rise in the activity of the secretory phospholipase A2 is the form enhancing oxidative stress and initiating inflammation [27–29]. Phospholipase C plays an important role in inflammatory signal activation and insulin resistance in human primary adipocytes [30]. Activation of phospholipase D facilitates oxidant release and redox regulation [31, 32]. Increased serum phospholipase D is associated with insulin resistance [33]. Uric acid is the major end product of purine metabolism [34]. It can act as either an antioxidant or a pro oxidant depending on the circumstances, especially depending on the availability of lipid hydroperoxides [35–38]. Lipid peroxidation injury is considered to be the reason for increased concentration in serum uric acid related to the pathogenesis of NAFLD [4, 36, 38, 39]. Increased systemic oxidative stress has long been recognized as an essential cause of HU and inflammation and as one of the important pathogeneses of NAFLD both in animal experiments and clinical studies [40–43]. Insulin resistance not only increases uric acid synthesis and reduces the renal excretion of uric acid but also promotes lipolysis of peripheral adipose tissue and increases free fatty acid influx into the liver, leading to NAFLD [44–47]. HU can contribute to the development of NAFLD via insulin resistance [48]. This study found that phosphatidic acid is up-regulated in serum during the progression from HU to HU+NAFLD, which suggests that oxidative stress and insulin resistance due to up-regulated phospholipase metabolism appear at least partially responsible for the development of steatosis in HU.

CE (18:0) is a cholesterol fatty acid ester and can be hydrolyzed by cholesterol esterase to produce cholesterol and free fatty acids. A high level of serum CE is associated with high insulin resistance [49]. CE is involved in LXR/RXR activation, which plays an important role in keeping the cholesterol balance outside and inside the cell [50]. LXR/RXR activation is known as one of the pathogeneses of NAFLD [51]. In this study, cholesteryl ester was up-regulated in the serums during the progression, implying that LXR/RXR activation is an important reason for the steatosis development. Inosine is a precursor of uric acid. The findings showed that inosine is down-regulated but uric acid is up-regulated in the serums of both HU and HU+ NAFLD. Lowered inosine may reflect a physiological compensatory mechanism counteracting increased uric acid to maintain the homeostasis of an organism. Perhaps serum inosine may be only a marker of the progression and not etiologically important in the disease.

**Fig 8:**
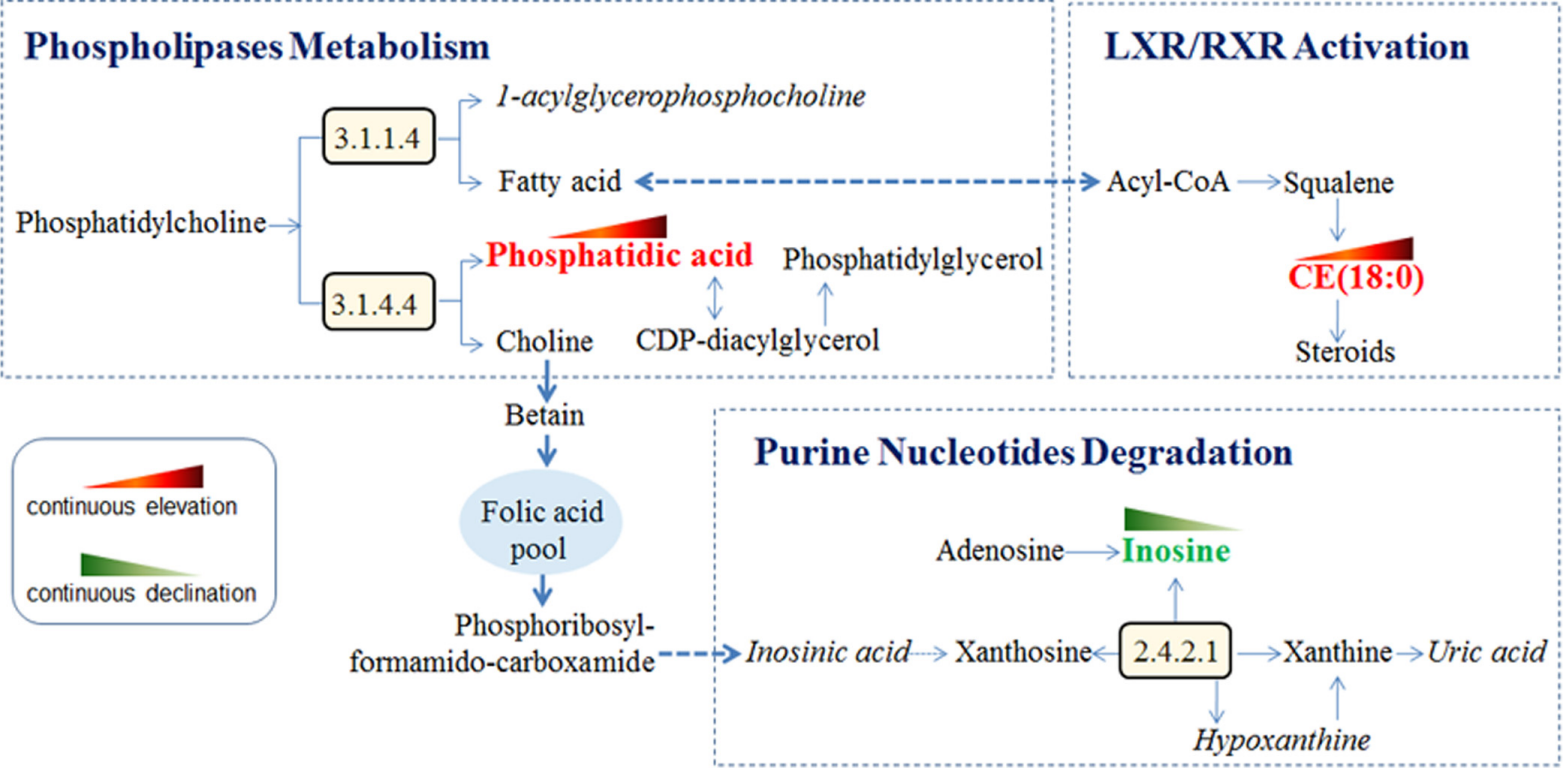
Perturbed metabolic regulatory network in response to the development of steatosis in HU. Up-regulated phosphatidic acid and CE (18:0) continue to rise; down-regulated inosine continues to fall. Accordingly, three pathways, phospholipase metabolism, purine nucleotide degradation and LXR/RXR activation, are perturbed in the steatosis development.

In conclusion, as shown in **Fig 8**, the development of steatosis in HU is characterized by up-regulated phosphatidic acid and CE (18:0) and down-regulated inosine. Perturbations of phospholipases, purine nucleotide degradation and LXR/RXR activation are found to be partially responsible for this development. A limitation of this study is that most of the research subjects were office workers; their prevalence of HU and HU+NAFLD may be higher than that in a rural area. It is necessary to expand the sample size in further studies.

## Supporting Information

S1 Fig Multiple pattern recognition of metabolites in Control and HU. (PDF)

S2 Fig Multiple pattern recognition of metabolites in Control and HU+NAFLD. (PDF)

S3 Fig Multiple pattern recognition of metabolites in initial HU and outcome HU+NAFLD. (PDF)

S1 Table Pathways associated with identified metabolites in HU. (PDF)

S2 Table Pathways associated with identified metabolites in HU+NAFLD. (PDF)

S3 Table Pathways associated with the metabolites predicting the progression. (PDF)

S4 Table Pathways associated with identified metabolites in outcome HU+NAFLD vs. initial HU. (PDF)

## Abbreviations

AST: glutamate amino transaminase
ALT: alanine transaminase
BUN: blood urea nitrogen
BMI: body mass index
BPCs: typical base peak chromatograms
CE: cholesterol ester
CRE: creatinine
DBP: diastolic blood pressure
EICs: extracted ion chromatograms
EMI: enzyme-metabolite interaction
FBG: fasting blood glucose
HDL: high-density lipoprotein cholesterol
HU: hyperuricemia
HU+NAFLD: hyperuricemia combined with non-alcoholic fatty liver disease
IPA: ingenuity pathway analysis
LDL: low-density lipoprotein cholesterol
LXR/RXR: liver X receptor/retinoid X receptor
MS: metabolic syndrome
NAFLD: non-alcoholic fatty liver disease
PCA: principal components analysis
PLS-DA: partial least squares discriminant analysis
PPI: protein-protein interaction
RSDs: relative standard derivations
Subjects with HU→HU+NAFLD: subjects who suffered from hyperuricemia first and one year later from HU+NAFLD
SBP: systolic blood pressure
SUA: serum uric acid
TG: triglyceride
TC: total cholesterol
