# Supplementary material for: The Potential Biomarkers to Identify the Development of Steatosis in Hyperuricemia
Source: PLoS One. 2016 Feb 18;11(2):e0149043. doi: 10.1371/journal.pone.0149043 (PMC4758628; doi:10.1371/journal.pone.0149043)
Supplement: S1 Fig — (PDF) [file pone.0149043.s001.pdf]

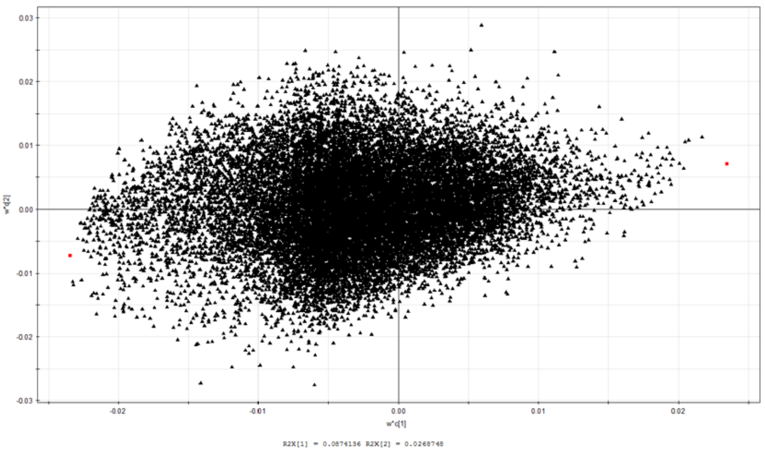

S1 Fig. Multiple pattern recognition of metabolites in Control and HU. PLS-DA loading plot (n = 49). Each triangle in the plot represents an ion. Ions far away from origin were responsible for potential biomarkers.
