# Supplementary material for: The Potential Biomarkers to Identify the Development of Steatosis in Hyperuricemia
Source: PLoS One. 2016 Feb 18;11(2):e0149043. doi: 10.1371/journal.pone.0149043 (PMC4758628; doi:10.1371/journal.pone.0149043)
Supplement: S2 Fig — (PDF) [file pone.0149043.s002.pdf]

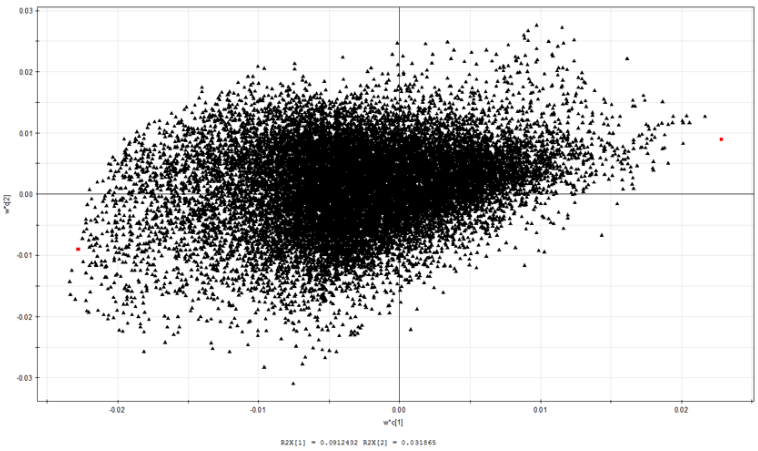


**S2 Fig.** Multiple pattern recognition of metabolites in Control and HU+NAFLD. PLS-DA loading plot (n = 69). Each triangle in the plot represents an ion. Ions far away from origin were responsible for potential biomarkers.
