# Supplementary material for: The Potential Biomarkers to Identify the Development of Steatosis in Hyperuricemia
Source: PLoS One. 2016 Feb 18;11(2):e0149043. doi: 10.1371/journal.pone.0149043 (PMC4758628; doi:10.1371/journal.pone.0149043)
Supplement: S3 Fig — (PDF) [file pone.0149043.s003.pdf]

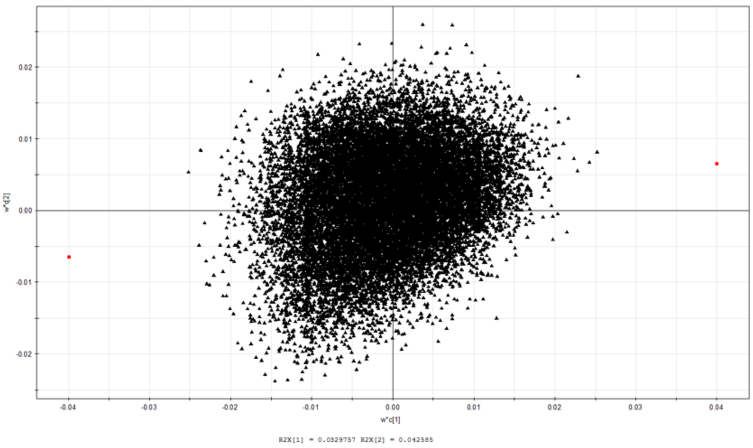

S3 Fig. Multiple pattern recognition of metabolites in initial HU and outcome HU+NAFLD. PLS-DA plot (n = 40). Each triangle in the plot represents an ion. Ions far away from origin were responsible for potential biomarkers.
