# Supplementary material for: The Potential Biomarkers to Identify the Development of Steatosis in Hyperuricemia
Source: PLoS One. 2016 Feb 18;11(2):e0149043. doi: 10.1371/journal.pone.0149043 (PMC4758628; doi:10.1371/journal.pone.0149043)
Supplement: S1 Table — (PDF) [file pone.0149043.s004.pdf]

S1 Table. Pathways associated with identified metabolites in HU.

N	Canonical Pathways	Molecules
1	Purine Nucleotides Degradation II	inosine, uric acid
2	LXR/RXR Activation	CE (18:0)
3	Phospholipases	phosphatidic acid
4	Serotonin Receptor Signaling	5-hydroxyindoleacetic acid
5	Purine Nucleotides De Novo Biosynthesis II	5-aminoimidazole ribotide
6	Valine Degradation I	L-valine
7	Noradrenaline and Adrenaline Degradation	3,4-dihydroxyphenylglycol
8	γ-glutamyl Cycle	pyrrolidonecarboxylic acid
