# Supplementary material for: The Potential Biomarkers to Identify the Development of Steatosis in Hyperuricemia
Source: PLoS One. 2016 Feb 18;11(2):e0149043. doi: 10.1371/journal.pone.0149043 (PMC4758628; doi:10.1371/journal.pone.0149043)
Supplement: S2 Table — (PDF) [file pone.0149043.s005.pdf]

S2 Table. Pathways associated with identified metabolites in HU+NAFLD.

N	Canonical Pathways	Molecules
1	Purine Nucleotides Degradation II	inosine, uric acid, inosinic acid
2	Serotonin Receptor Signaling	5-hydroxyindoleacetic acid, L-tryptophan
3	Tryptophan Degradation III	kynurenine, L-tryptophan
4	Phospholipases	phosphatidic acid
5	LXR/RXR Activation	CE (18:0)
6	Methylglyoxal Degradation I	D-lactic acid
7	γ-glutamyl Cycle	pyrrolidonecarboxylic acid
8	Melatonin Degradation II	5-methoxyindoleacetic acid
9	Tyrosine Degradation I	4-fumarylacetoacetate
10	Purine Nucleotides De Novo Biosynthesis II	Inosinic acid
