# Supplementary material for: The Potential Biomarkers to Identify the Development of Steatosis in Hyperuricemia
Source: PLoS One. 2016 Feb 18;11(2):e0149043. doi: 10.1371/journal.pone.0149043 (PMC4758628; doi:10.1371/journal.pone.0149043)
Supplement: S3 Table — (PDF) [file pone.0149043.s006.pdf]

S3 Table. Pathways associated with identified metabolites predicting the progression.

N	Canonical Pathways	Molecules
1	Purine Nucleotides Degradation II	Inosine, uric acid, inosinic acid
2	Tryptophan Degradation III	kynurenine, L-tryptophan
3	Serotonin Receptor Signaling	5-hydroxyindoleacetic acid, L-tryptophan
4	Purine Nucleotides De Novo Biosynthesis II	5-aminoimidazole ribotide, inosinic acid
5	Phospholipases	phosphatidic acid
6	LXR/RXR Activation	CE (18:0)
7	Tyrosine Degradation I	4-fumarylacetoacetate
8	Methylglyoxal Degradation I	D-lactic acid
9	γ-glutamyl Cycle	pyrrolidonecarboxylic acid
10	Valine Degradation I	L-valine
11	Noradrenaline and Adrenaline Degradation	3,4-dihydroxyphenylglycol
