# Supplementary material for: The Potential Biomarkers to Identify the Development of Steatosis in Hyperuricemia
Source: PLoS One. 2016 Feb 18;11(2):e0149043. doi: 10.1371/journal.pone.0149043 (PMC4758628; doi:10.1371/journal.pone.0149043)
Supplement: S4 Table — (PDF) [file pone.0149043.s007.pdf]

S4 Table. Pathways associated with identified metabolites in outcome HU+NAFLD vs. initial HU.

N	Canonical Pathways	Molecules
1	Phospholipases	1-acylglycerophosphocholine, phosphatidic acid
2	Purine Nucleotides Degradation II	inosine, hypoxanthine
3	LXR/RXR Activation	CE (18:0)
4	Lipoate Biosynthesis and Incorporation II	octanoic acid
5	Histidine Degradation VI	urocanic acid
6	Tyrosine Degradation I	4-fumarylacetoacetate
7	Leukotriene Biosynthesis	leukotriene A4
8	Lysine Degradation V	pipecolic acid
